# Evaluation of a Fully Automated Research Prototype for the Immediate Identification of Microorganisms from Positive Blood Cultures under Clinical Conditions

**DOI:** 10.1128/mBio.00491-16

**Published:** 2016-04-19

**Authors:** Jay M. Hyman, John D. Walsh, Christopher Ronsick, Mark Wilson, Kevin C. Hazen, Larisa Borzhemskaya, John Link, Bradford Clay, Michael Ullery, Mirta Sanchez-Illan, Steven Rothenberg, Ron Robinson, Alex van Belkum, W. Michael Dunne

**Affiliations:** a bioMérieux Inc., Durham, North Carolina, USA; b Department of Pathology, Clinical Microbiology Laboratory, Duke University, Durham, North Carolina, USA; c bioMérieux SA, La Balme les Grottes, France; d bioMérieux Inc., Hazelwood, Missouri, USA

## Abstract

A clinical laboratory evaluation of an intrinsic fluorescence spectroscopy (IFS)-based identification system paired to a BacT/Alert Virtuo microbial detection system (bioMérieux, Inc., Durham, NC) was performed to assess the potential for fully automated identification of positive blood cultures. The prototype IFS system incorporates a novel method combining a simple microbial purification procedure with rapid *in situ* identification via spectroscopy. Results were available within 15 min of a bottle signaling positive and required no manual intervention. Among cultures positive for organisms contained within the database and producing acceptable spectra, 75 of 88 (85.2%) and 79 of 88 (89.8%) were correctly identified to the species and genus level, respectively. These results are similar to the performance of existing rapid methods.

## IMPORTANCE

A fully automated research platform was developed to identify microbial growth from positive blood cultures in <15 min. Because of the automated format, results can be generated during all shifts, with or without staffing, which in turn could promote more timely administration of target antimicrobial therapy.

## INTRODUCTION

The recovery of microorganisms from liquid cultures of blood represents one of the most critical results generated by diagnostic clinical microbiology laboratories in terms of therapeutic patient management and overall management of hospital-based antibiotic usage. Prompt reporting of Gram stain results on positive blood cultures is essential to patient outcome (1). It is also likely that additional details concerning an organism’s identity beyond the level of Gram stain, but reported within the same time frame, would allow for a more targeted approach toward the selection of empirically based therapy prior to availability of antimicrobial susceptibility test (AST) results (2). In a recent proof-of-principle study, Walsh et al. (3) demonstrated that the combined use of a selective lysis buffer, an aqueous density cushion in an optical microcentrifuge tube, and an intrinsic fluorescence spectroscopy system (IFS) paired with a reference spectral database allowed for the correct identification of 96.5% of 1,121 single-organism-spiked positive blood culture samples to the species level. Among the total number of strains tested, there were 9 incorrect identifications (0.8%), but 8 of 9 of these discordant calls were directed to species within the correct genus. In the next phase of IFS development as a diagnostic tool, a fully automated research prototype was constructed which allowed for real-time (<15 min) identification of microorganisms from a positive blood culture bottle without requiring any user intervention. To determine the performance characteristics of this approach with actual clinical samples and under working laboratory conditions, a prototype system was evaluated in a major medical center clinical microbiology laboratory over a period of 28 weeks, and the results are presented here. 

(Presented in part at the American Society for Microbiology 115th General Meeting, New Orleans, LA, 30 May to 2 June 2015, poster 1093.)

## RESULTS

During the 7-month course of this study, a total of 2,781 blood cultures were evaluated, and 132 of these signaled positive via the Virtuo system. One hundred twenty-three were determined to be true positive cultures and of these, 113 (91.9%) were processed by the IFS (100 single-organism and 13 mixed-culture samples) (Table 1). The prototype IFS performed consistently over this trial period, with the only maintenance required being periodic replacement of the spectrophotometer lamp. Nine of the 132 cultures were classified as false-positive results (overall false-positivity rate, 0.32%). None of the false-positive cultures demonstrated a column of cells in the optical capillary after centrifugation, produced a fluorescence spectrum, or generated colonies on subculture. Ninety of the 113 analyzable cultures (77 single-organism and 13 mixed-culture samples) yielded species that were represented within the database either as the sole or predominant organism (Tables 1 and 2), while 23 cultures grew species that were not in the database or were not identified by the Vitek mass spectrometry (MS) system (Table 2). Spectra from two cultures showed system anomalies that made interpretation unreliable. Therefore, 88 of the 90 cultures that contained organisms represented in the spectral database could be analyzed. Of these, 75 (85.2%) were identified to the species level, concordant with matrix-assisted laser desorption ionization–time of flight MS (MALDI-TOF MS) identification, while 79 (89.8%) were concordant to genus-level identification. For single-organism cultures, 87% of Gram-positive and 78% of Gram-negative isolates were correctly identified to the species level (Tables 1 and 3). The one yeast recovered (*Candida tropicalis*) was correctly identified. Of the 13 mixed-microorganism cultures, two distinct organisms were identified from one, based on different IF spectra and differential sedimentation rates of cells within the quartz capillary (see below). A single identification of the dominant organism was generated for 11 of the remaining 12 mixed cultures (Tables 1 and 2).

**TABLE 1:** Comparison of identification of positive blood culture isolates via IFS versus MALDI-TOF MS

IFS/MALDI-TOF result	No. of cultures^a^	Concordant species ID (no. [%]) based on^b^:				Concordant genus ID (no. [%])
		One choice	Low	Overall	Discordant or no species ID	
ID via IFS database						
Single isolate	77	62 (80.5)	1 (1.1)	63 (81.8)	14 (18.2)	
Mixed culture	13	12 (92.3)	0	12 (92.3)	1 (7.7)	
Combined^c^	90	74 (82.2)	1 (1.1)	75 (83.3)	15 (16.7)	79 (87.8)
ID via IFS database and acceptable spectra				75 (85.2)	13 (14.8)	79 (89.8)

a A total of 113 cultures were analyzed for identification (ID) of the species or genus.

b Species identifications are reported for comparisons based on one choice, low discriminatory power (“low”), or both (overall).

c “Combined” data are the totals for both the single-isolate and mixed cultures.

**TABLE 2:** Species identified from single-isolate or mixed cultures and Gram stain results for culture-positive bottles obtained during the preclinical trial of the IFS

Gram stain result^a^	Microorganism(s)^b^	No. of isolates
GNR	*Citrobacter freundii*	1
Yeast	*Candida tropicalis*	1
GNR	*Enterobacter cloacae*	3
GNR	*Escherichia coli*	10
GPC	*Enterococcus faecium*	7
GNR	*Klebsiella pneumoniae*	8
GNR	*Pseudomonas aeruginosa*	4
GPC	*Staphylococcus aureus*	18
GPC	*Staphylococcus epidermidis*	14
GPC	***Staphylococcus hominis***	3
GPC	***Staphylococcus lugdunensis***	2
GPC	*Staphylococcus capitis*	1
GNR	*Stenotrophomonas maltophilia*	3
GPC	***Streptococcus mitis/S. oralis***	2
GPR and GNR	***Acinetobacter baumannii* Cpx*/E. cloacae***	1
GNR	***C. freundii/Klebsiella oxytoca***	1
GPC	***Enterococcus faecalis/S. epidermidis***	1
GPC	*E. faecium*/*A. xylosoxidans*	1
GPC and GNR	***E. faecium/K. pneumoniae***	1
GNR and GPC	*K. pneumoniae/E. coli/E. faecium*/ *Citrobacter youngii*	1
GPC and GNR	***K. pneumoniae/E. faecium***	1
GNR	***K. pneumoniae/P. aeruginosa***	1
GPC and GNR	***K. pneumoniae/S. agalactiae***	1
GPC and GNR	***K. pneumoniae/S. agalactiae/S. aureus***	1
GPC and GNR	***K. pneumoniae/S. aureus***	1
GPC and GNR	***S. maltophilia/E. faecium***	1
Mixed GNR	*S. maltophilia***/***Pseudomonas putida*	1
GPC	*Abiotrophia defectiva*	2
GNR	*Achromobacter xylosoxidans*	6
GNR	*Burkholderia cepacia* complex	2
GPR	*Bacillus* spp., not *Bacillus anthracis*	2
GNR	*Citrobacter amalonaticus*	1
Yeast	*Cryptococcus neoformans*	1
GPR	*Lactobacillus* sp*.*	1
GPC	*Micrococcus luteus*	2
GPR	*Mycobacterium mucogenicum* group	1
GPC	*Staphylococcus auricularis*	1
GPC	*Staphylococcus haemolyticus*	1
GPC	*Streptococcus salivarius*	1
GPR and GNR	No ID by Vitek-MS	2

a GNR, gram-negative rods; GPC, gram-positive cocci; GPR, gram-positive rods.

b Species identifications were determined via MALDI-TOF MS. Those species represented in the IFS database developed for the study are highlighted in bold.

## DISCUSSION

We have demonstrated here the potential to use intrinsic fluorescence spectroscopy combined with a fully automated robotic processing platform to rapidly (≤15 min) identify microbial growth from a positive blood culture without any human intervention. While rapid identification of an organism from a positive blood culture does not ensure appropriate antimicrobial therapy in the absence of susceptibility results, it would (when combined with local or regional antibiogram data) likely improve the selection of suitable therapeutic choices and potentially reduce unnecessary use of inappropriate drugs. Of the 100 positive blood cultures in this study that generated a single organism on subculture, 77 of the isolates were represented within the database developed for this study, while 23 were not. Among the “out-of-model” species in this study, *Achromobacter xylosoxidans* (*n* = 6) was the most frequent species isolated. Less frequent isolates recovered at this large teaching hospital included *Abiotrophia defectiva*, *Burkholderia cepacia*, *Cryptococcus neoformans*, *Citrobacter amalonaticus*, and the *Mycobacterium mucogenicum* group. Smaller hospitals or those without transplantation services would likely have a higher percentage of isolates within the preliminary 37-species database reported here. However, the IFS database does have the capability to be expanded to reflect these and other organisms not currently represented but identified by regional and/or national epidemiological data.

The automated blood culture identification results reported here for this early-stage prototype system (85% correct to the species level, 90% correct to the genus level) are on par with many previously reported MALDI-TOF study results (summarized in reference 3), but our results were generated in real time and without any user intervention. For studies utilizing molecular methods, higher accuracies directly from blood cultures have been reported (4–6), but such methods are expensive and require manual intervention.

In addition to the overall findings of the study, three highlights are worth mentioning. First, the automated IFS identified 17 of 18 cultures positive for *Staphyloccos aureus* (Table 3) and 10 of 11 patients with *S. aureus* bacteremia. This included cultures that signaled positive during all three traditional shifts of laboratory staffing. The advantage of this system for microbiology laboratories that cannot provide continuous 24-h support on so-called off-shifts is obvious if results can be conveyed directly to clinical staff. The second highlight involves a blood culture in which the Gram stain of the growth was ambiguous, i.e., interpreted to show mixed Gram-positive and Gram-negative coccobacilli. The IFS identified *Acinetobacter baumannii* from the positive bottle*—*an organism notorious for variable Gram stain results. Subcultures of the bottle produced primarily *A. baumannii* with rare mixed colonies of *Enterobacter cloacae*, but no Gram-positive organisms. Finally, one culture generated two distinct identifications via the IFS. Optic fibers 4 to 6 gave a preliminary identification of *Klebsiella/Enterobacter* spp., while fibers 7 and 8 provided an identification of *Staphylococcus* spp. with a high probability of *S. aureus*. Subcultures produced mixed colonies of *Klebsiella pneumoniae* and *S. aureus*, which was confirmed from a second positive blood culture from this patient. The latter finding was made possible by switching from a bottom-read optical system, as described previously (3), to a side-read system in which 8 optical fibers positioned along the side of the quartz capillary could query the entire length of the cell mass column, where differential sedimentation of mixed organisms could be recognized.

**TABLE 3:** Correct identifications of species represented in the IFS database among 75 monomicrobic cultures

Gram stain and MALDI-TOF identification	Total no. of cultures	No. (%) correctly identified to species level by the IFS
Gram positive		
* Staphylococcus aureus*	18	17 (94)
* S. epidermidis*	14	13 (93)
* S. hominis*	3	1 (33)
* S. lugdunensis*	2	2 (100)
* S. capitis*	1	0 (0)
* Enterococcus faecium*	7	6 (86)
* Streptococcus mitis/S. oralis*	2	2 (100)
Total for Gram positives	47	41 (87)
Gram negative		
* Escherichia coli*	8	6 (75)
* Klebsiella pneumoniae*	8	4 (50)
* Pseudomonas aeruginosa*	4	4 (100)
*Enterobacter cloacae* complex	3	3 (100)
* Stenotrophomonas maltophilia*	3	3 (100)
* Citrobacter freundii*	1	1 (100)
Total for Gram negatives	27	21 (78)
Fungal species		
* Candida tropicalis*	1	1 (100)
All organisms	75	63 (84)

While other systems have been developed for the purpose of rapid identification of growth obtained from positive blood cultures, including the BCID FilmArray (BioFire Diagnostics, Salt Lake City, UT), Nanosphere’s Verigene system (Northbrook, IL), and various MALDI-TOF MS applications, most require some degree of manual processing and generate results within 1 to 3 h (4–6). This report is the first to describe a system that requires no manual intervention when paired with a Virtuo blood culture system and provides a result in less than 15 min after a blood culture signals positive. Additional maturation of the IFS database would allow for expansion of the “in-model” list of species, including less-frequently recovered organisms and potential contaminants, and thus increase the likelihood of single-choice species identification rates above the observed 85% reported here. In a recent report scrutinizing the effectiveness of clinical microbiology practices intended to decrease the time of switching to targeted antimicrobial therapy in patients with bloodstream infections, it was noted that “rapid identification methods may have multiple positive impacts on patient outcomes, including reductions in mortality, morbidity, hospital LOS [length of stay], antibiotic use, and health care expenses” (7). If this assumption proves to be correct, when paired with rapid direct reporting strategies, the IFS or systems like it have the potential to provide value beyond the simplicity of fully automated identification of organisms from positive blood culture bottles.

## MATERIALS AND METHODS

**FIG 1:**
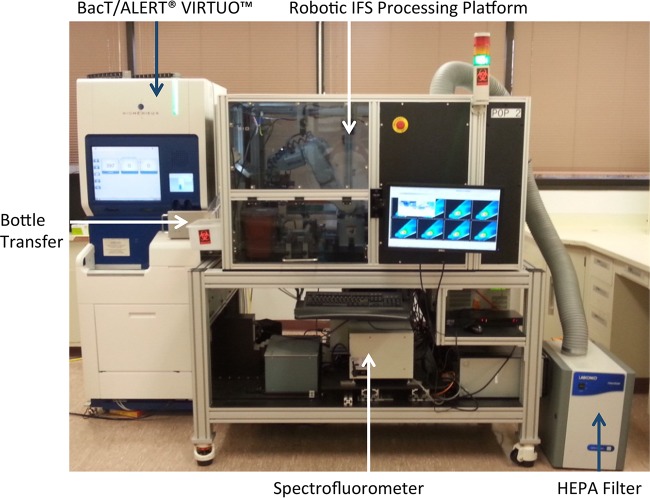
Prototype IFS, consisting of a Virtuo blood culture unit, a robotic bottle-processing station, a spectrofluorometer, and a HEPA filtration system. Positive blood culture bottles were transferred from the Virtuo system to the processing station via a linear slide mechanism.

**FIG 2:**
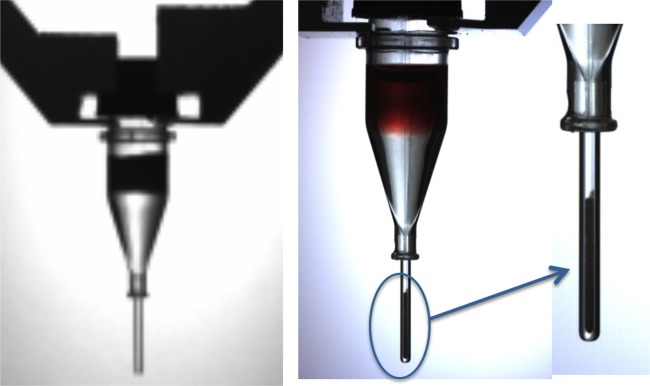
Optical microcentrifuge tube (left panel) containing the density buffer and a quartz capillary tube attached to the bottom of an acrylic body. After layering the blood culture lysate and centrifugation, the cell mass is collected in the capillary tube at the bottom (center and right panels), where intrinsic fluorescence analysis occurs.

The prototype IFS module consisted of a six-axis robotic unit housed within an enclosure equipped with a HEPA filtration unit (Fig. 1). The module also contained a computer-controlled microcentrifuge with a custom-built bucket and swing-out rotor, a vortex mixer, and an optical reading station attached to a fluorescent spectrophotometer via a custom fiber optic interface, a sharps container, a user interface display screen, and all necessary disposables to complete the identification process. The IFS module adjoined a BacT/Alert Virtuo blood culture system (bioMérieux, Inc., Durham, NC) with a linear slide allowing for the automatic transfer of positive bottles to the IFS. Once inside the robotic processing unit, the bottle was vented and 1.0 ml of broth was transferred from the bottle via a specially designed disposable syringe to a prewarmed tube (37°C) containing lysis buffer. The lysis buffer has been described previously (3). The tube was further incubated at 37°C for 30 s and vortexed for 5 s. Lysate was then removed from the lysis tube and added to an optical microcentrifuge tube containing a density buffer and a quartz capillary attached to the bottom of an acrylic body (Fig. 2). The density buffer has also been described previously (3). The syringe was disposed into a sharps container, and the optical centrifuge tube was transferred to the microcentrifuge and spun at 10,600 × *g* for 2 min, which effectively generated a column of microbial cells in the optical capillary portion of the centrifuge tube (Fig. 2). The quartz capillary was inspected by a robotic vision system to ensure the presence of a recovered column of cells and to prevent further processing if the capillary was cracked (the entire system would shut down, except the HEPA filtration unit, for decontamination if such an event occurred). Several vision analysis stations were employed within the IFS to confirm critical processing steps and guide the positioning of disposables.

**FIG 3:**
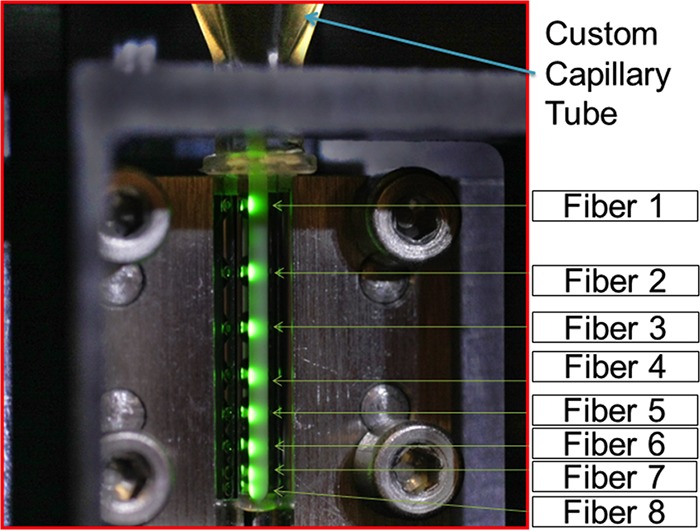
Optical capillary tube in the reading station, showing the positions of the 8 optical fibers used to collect spectra along the length of the quartz capillary tube containing the concentrated biomass of microbial growth from the lysed and centrifuged blood culture sample.

The processed tube was then placed in a reader station, where 8 optical fibers collected fluorescent spectra spaced vertically along the entire length of the capillary tube to provide additional opportunities to enhance the accuracy of the identification system, including estimation of total cell mass and determination of redundancy of spectral measurements and also to detect stratification of different cell populations in mixed cultures due to potential differences in sedimentation rates and buoyant densities (Fig. 3). This arrangement also allowed debris that collected at the bottom of the tube to be ignored. The entire automated process, from reading a positive bottle’s bar code to collection of spectra, took 12 min to complete. The on-board fluorescence spectrophotometer performed an automatic calibration daily to ensure wavelength accuracy and sufficient lamp intensity. Once a day, lab personnel filled color-coded trays with the three disposables (venting/syringe unit, lysis tube, and optical microcentrifuge tube).

**FIG 4:**
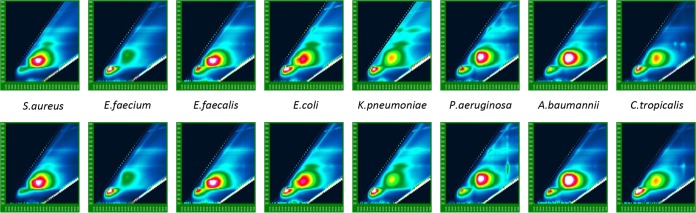
Comparison of typical intrinsic fluorescence spectra obtained with seeded samples (top) and clinical positive blood cultures (bottom).

The database used for identification in this study was developed using 751 seeded blood cultures consisting of 37 species and 575 strains. The excitation scan range was every 5 nm, from 260 to 600 nm, and the emission range was up to 2 times the excitation range. Approximately 6,000 usable excitation-emission pairs/spectra were collected. Two hundred fifteen excitation-emission pairs were selected for discriminant analysis using custom software to visualize spectral differences between species (Fig. 4). Finally, a classification model was optimized to 94 excitation-emission pairs by using forward stepwise discriminant analysis with venetian blind cross-validation. Spectra of the clinical cultures were analyzed off-line as unknowns using the discriminant function generated by the seeded culture database. Identification was determined using posterior probabilities of class (in this case, species) membership. Any probability of ≥80% was considered a one-choice result, 20 to 80% was considered low discrimination, and a probability of ≤20% was considered incorrect. This analysis did not attempt to determine goodness of fit, so the results “incorrect” and “no identification” were indistinguishable.

**FIG 5:**
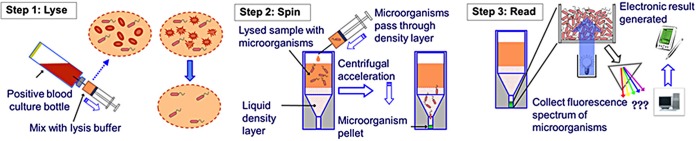
Summary of the overall lyse, spin, and read process orchestrated by the robotic processing station.

The entire lyse, spin, and read process is summarized in Fig. 5.

The study protocol was approved by the Duke University Medical Center (Durham, NC) institutional review board (IRB) prior to initiation. A single BacT/Alert standard aerobic (SA) bottle was collected in addition to the standard blood culture set used at the medical center, and the volume of blood introduced in that bottle was recorded. The study bottle was then loaded into a prototype version of the Virtuo blood culture system and monitored for a period of 5 days before automatically being discarded as negative. Positive bottles were processed by the IFS, and the results were recorded. In addition, the contents of positive bottles were used to prepare Gram stains and subcultures for reference identification of isolates using the Vitek MS MALDI-TOF system (bioMérieux, Inc.). Approximately 5 ml of the positive broth was transferred to a sterile screw-cap tube and stored at −70°C for any further evaluations. If Gram staining and subculture could not be performed immediately, bottles could be stored at refrigerator temperatures for up to 48 h prior to processing.
