# Author Correction for Hyman et al., Evaluation of a Fully Automated Research Prototype for the Immediate Identification of Microorganisms from Positive Blood Cultures under Clinical Conditions

**DOI:** 10.1128/mBio.00705-16

**Published:** 2016-05-24

**Authors:** Jay M. Hyman, John D. Walsh, Christopher Ronsick, Mark Wilson, Kevin C. Hazen, Larisa Borzhemskaya, John Link, Bradford Clay, Michael Ullery, Mirta Sanchez-Illan, Steven Rothenberg, Ron Robinson, Alex van Belkum, W. Michael Dunne

**Affiliations:** a bioMérieux Inc., Durham, North Carolina, USA; b Department of Pathology, Clinical Microbiology Laboratory, Duke University, Durham, North Carolina, USA; c bioMérieux SA, La Balme les Grottes, France; d bioMérieux Inc., Hazelwood, Missouri, USA

## AUTHOR CORRECTION

Volume 7, no. 2, doi:10.1128/mBio.00491-16, 2016. The following conflict of interest statement should be added:

All authors except K.C.H. are or have been employees of bioMérieux, Inc. (L.B. has since retired). We have no other conflicts of interest to declare.

